# Risk factors for repeated emergency compulsory psychiatric admissions

**DOI:** 10.1192/bjo.2020.153

**Published:** 2020-12-22

**Authors:** Mark H. de Jong, André I. Wierdsma, Jeroen Zoeteman, Christina A. van Boeijen, Arthur R. Van Gool, Cornelis L. Mulder

**Affiliations:** Yulius Mental Health, the Netherlands; Epidemiological and Social Psychiatric Research Institute (ESPRi), Department of Psychiatry, Erasmus University Medical Centre, the Netherlands; Spoedeisende Psychiatrie Amsterdam, the Netherlands; GGNet, the Netherlands; Emergis, the Netherlands; Epidemiological and Social Psychiatric Research Institute (ESPRi), Department of Psychiatry, Erasmus University Medical Centre, the Netherlands

**Keywords:** Emergency psychiatry, compulsory admission, coercion, self-neglect, functioning

## Abstract

**Background:**

The characteristics of patients who have repeated compulsory psychiatric admissions are largely unknown.

**Aims:**

To investigate the frequency and risk factors for repeated emergency compulsory psychiatric admission (ECPA); and to identify targets for interventions to reduce repeated ECPA.

**Method:**

Data were collected from a database of electronic patient files (EPFs) held by three psychiatric emergency services (PES) in the Netherlands. Analyses were based on the data for adult patients (aged 18–75 years) with a first PES contact in 2010–2015. Using descriptive statistics and regression analysis, we studied the associations between baseline patient factors and repeated ECPA and time to readmission, within a 2-year follow-up period.

**Results:**

We included 6059 patients: 15.6% had two or more ECPAs. In total, 66% of second ECPAs had occurred within 6 months of the first. About 30% of all ECPAs were repeated ECPAs. Two baseline factors were associated with a higher frequency of a second ECPA: history of receiving any mental healthcare treatment, whether in-patient or out-patient or both, and a lower level of self-care. Three were associated with a lower frequency: ethnicity (other than Dutch), older age and suicidality. Lower Global Assessment of Functioning (GAF) scores and housing problems were associated with a shorter time to compulsory readmission and persistent psychiatric problems with a longer time to compulsory readmission.

**Conclusions:**

We found that 15.6% of patients had two or more ECPAs. Two-thirds of the second ECPAs had occurred within 6 months of the first. Like earlier studies, the risk factors we identified suggest that interventions to reduce the risk of repeated compulsory psychiatric admission should seek to improve self-care, general daily functioning and homelessness.

## Background

Compulsory psychiatric admissions are very stressful events in the lives of patients and their caregivers.^1^ A recent systematic review stated that the greatest risks for such admissions were associated with previous involuntary admissions to hospital and a diagnosis of a psychotic disorder, and to a lesser extent with being male, single and unemployed, receiving welfare benefits and having a diagnosis of bipolar disorder.^2^ The unfortunate fact that many patients undergo repeated compulsory admissions, is indicative of a need to develop targeted interventions.

Most of the little research that has been done on ‘revolving-door’ patients has focused on voluntary admissions.^3–7^ It found four predictors of frequent readmissions: a history of previous psychiatric admissions, a diagnosis on the psychosis spectrum, being unemployed and living in residential accommodation. But few studies have investigated the prevalence of repeated compulsory psychiatric admissions and their risk factors. During a 5-year follow-up period, a Dutch study found that the frequency of such admissions was 37%.^8^ Compulsory readmission was associated with greater consumption of care in the 5 years before inclusion, a history of compulsory psychiatric admissions, younger age and living alone. During a 7-year follow-up period, a retrospective Taiwanese study in patients with schizophrenia found a frequency of repeated compulsory psychiatric admissions of 5.5%, and that the risk of compulsory readmission was higher in patients with a prior compulsory admission than in those whose prior admission had been voluntary (adjusted hazard ratio (HR) = 1.31).^9^ Finally, a prospective study in Switzerland with a 24-month follow-up period found a frequency of repeated compulsory psychiatric admissions of 36% and that two factors were associated with such admissions: previous compulsory admission – especially when it had been because of endangerment of others – and a diagnosis on the psychosis spectrum.^10^

One important reason for performing these studies is to identify risk factors that may be modifiable targets for interventions intended to reduce repeated compulsory psychiatric admissions. Two of the studies referred to above identified two such risk factors in two different countries: living alone and endangerment of others. As it might be possible to relate these findings to regional variations, it is important to investigate whether they can be replicated in another country.

## Purpose of the study

In our study we therefore focused on identifying targets for interventions intended to reduce repeated emergency compulsory psychiatric admissions (ECPA). Our specific aims were to investigate the following: the frequency of repeated ECPAs; the associations between clinical, demographic and process factors and the risk of repeated ECPA; and the associations between these same factors and the time to compulsory readmission.

## Method

### Setting

Our research included patients using psychiatric emergency services (PES) in three Dutch cities: Amsterdam, Apeldoorn and Rotterdam. Whereas Amsterdam and Rotterdam are large cities with extensive suburbs, Apeldoorn is relatively small and has rural surroundings. All three PES are part of an integrated mental healthcare institution in their particular geographic areas. Patients are usually referred by general practitioners, police or physicians in the general hospital.

The three PES involved in the study assessed the patients wherever they were: at home, in the emergency room at a general hospital, at the police station, in the offices of the mental health services or sometimes in an emergency room specially intended for psychiatric crisis care. The crisis assessments were made by a psychiatrist (or a psychiatry resident) and a nurse. After the assessment, the patients were transferred for admission – voluntary or compulsory – or out-patient follow-up or they received no aftercare. To promote the standardisation and uniform registration of all psychiatric crises, they used a concise web-based electronic patient file (EPF) specially designed for acute psychiatric care. Our study is based on the data from these EPFs.

### Data and materials

The EPF data were available from the inception of the database in 2008 up to the end of 2017. Initial inspection of the data showed that the vast majority of second ECPAs had occurred within 2 years of the index ECPA. To create corresponding baseline positions and follow-up periods for all patients included in the analysis, we chose to include unique patients who had no emergency contact in the first 2 years of the database (2008 and 2009) and could be followed for 2 years. We therefore based all our analyses on data from patients whose first PES contact had taken place between 2010 and the end of 2015.

Completing EPFs was part of the daily routine at the three PES. Entries included the patients’ demographic and clinical characteristics, such as gender, age, living situation, homelessness, diagnostic category, substance misuse, Global Assessment of Functioning score (GAF)^11^ and the Severity of Psychiatric Illness (SPI) rating scale.^12^ The latter is an instrument for exploring the patient's problems in terms of (a) problem severity; (b) psychiatric complications and comorbidity; and (c) complications in the treatment process. Each item is scored on a four-point scale, ranging from no risk or symptoms, through slight and moderate symptoms to high risk or severe symptoms. The Appendix shows the items that are included on the SPI.

The EPF is also used to register basic process information, including the time at which the contact with the patient took place, the referrer and the type of intervention, i.e. voluntary or compulsory admission, or no admission. To ensure acceptable accuracy with regard to preventing double registrations of the same patient, all patient data were carefully anonymised and a unique EPF identification number was attributed.

The authors assert that all procedures contributing to this work comply with the ethical standards of the relevant national and institutional committees on human experimentation and with the Helsinki Declaration of 1975, as revised in 2008. All procedures involving patients were approved by the Medical Ethics Committee at the Erasmus University Medical Centre (approval number: MEC-2020-0441). Under Dutch legislation on healthcare in general and medical research with human patients in particular, patient file research does not require informed consent. The above-mentioned committee classified our study as lying outside the scope of the Netherlands’ Medical Research Involving Human Subjects Act, and confirmed that if the patient data was anonymised no informed consent would be required.

### Judicial aspects of compulsory psychiatric admissions

In the Netherlands, ECPA are intended for crises and acutely dangerous situations that require immediate admission (i.e. within 24 h). This procedure requires a mandatory psychiatric assessment by a psychiatrist. According to the legal requirements of an ECPA, a psychiatrist completes the standardised Mental Health Act form (also applicable during the research period) to report on psychiatric status and diagnosis, dangerousness, absence of consent for treatment and admission, and absence of any less radical – i.e. non-compulsory – intervention to avert the present danger. After completion of the mandatory procedure, the detention is sanctioned by the mayor. Within 5 working days, a judge reviews the mayor's decision in a court session in the psychiatric clinic.

### Statistical analysis

Descriptive statistics were used to summarise the demographic, clinical and contextual characteristics of all patients who had either a single ECPA or two or more ECPAs. Multiple logistic regression analysis was used to identify variables associated with repeated ECPA, and Cox regression analysis to explore variations in the time to compulsory readmission. As well as patient characteristics and process factors, each SPI item was separately entered into the model. Following Hosmer & Lemeshow, variable selection was based on a stepwise procedure with *P* < 0.20 as entry level and *P* > 0.05 as removal level.^13^ Odds ratio (OR) estimates and their corresponding 95% CIs were calculated for explanatory variables in the final models. All statistical analyses were performed using SPSS version 25.

## Results

### Patients

From 2010 through 2015, the three PES assessed a total of 27 186 patients (aged 18–75 years), 6059 of whom (22.3%) had one or more ECPAs. Table 1 summarises the patient characteristics of patients who had one ECPA and of those who had two or more ECPAs.

**Table 1:** Baseline characteristics of all patients with one emergency compulsory psychiatric admission (ECPA) and with two or more ECPAs (first ECPA 2010–2015)

	1 ECPA	≥ 2 ECPAs	Total
*n*	5114	945	6059
Gender, % male	61.0	62.0	61.1
Age, years: median (IR)	40 (23)	37 (23)	39 (23)
Ethnicity, % born Dutch^a^	76.0	79.7	76.6
Living with family, %^b^	36.1	38.8	36.6
Homeless, %	6.5	6.5	6.5
History of any mental healthcare, %	29.2	32.5	29.7
Psychotic disorder, primary diagnosis %	60.5	61.4	60.7
Personality disorder, primary diagnosis %	2.1	3.5	2.3
Any alcohol or drugs misuse, %^c^	62.6	65.5	63.0
SPI sum, mean (s.d.)	25.6 (5.6)	25.8 (5.3)	25.7 (5.6)
GAF score, mean (s.d.)	30.4 (9.2)	30.2 (9.0)	30.4 (9.2)

IR, interquartile range; SPI, Severity of Psychiatric Illness; GAF, Global Assessment of Functioning.

a. Data available for *n* = 4755.

b. Data available for *n* = 4652.

c. Data available for *n* = 4190.

### Frequency of ECPAs

In the group of 6059 patients who had been admitted compulsorily between 2010 and the end of 2015, 945 patients (15.6%) had two or more ECPAs within 2 years of the index ECPA. These 945 patients counted for nearly 30% of the total number of ECPAs during the observation period, 12.3% of them having had two ECPAs (accounting for 20.5% of the total number of ECPAs); 2.4% having had three ECPAs (6.1% of the total); and 0.8% having had four ECPAs (2.9% of the total). Two-thirds of all second ECPAs (66.1%) had occurred within 6 months of the first.

The small but interesting subgroup of patients who had ≥4 ECPAs during the observation period (*n* = 53) – who, unsurprisingly, represented the largest proportion of readmissions within 6 months (81.1%) – had several specific characteristics, representing the largest proportion of patients with a baseline history of receiving any psychiatric treatment, whether in-patient or out-patient or both (34.0%, *n* = 18), with a severe score on the SPI item suicidality (32.1%, *n* = 17), with a personality disorder (7.5%, *n* = 4), with alcohol or drugs misuse (71.8%, *n* = 38) and of those who were homeless (15.1%, *n* = 8).

### Factors associated with compulsory readmission

The following baseline factors were associated with a higher frequency of repeated ECPAs: history of receiving any psychiatric treatment, whether in-patient, or out-patient, or both (OR) = 1.23, 95% CI 1.05–1.44) and lower level of self-care (OR = 1.21, 95% CI 1.03–1.42). Baseline factors associated with a lower frequency of repeated ECPAs were ethnicity (other than Dutch) (OR = 0.71, 95% CI 0.59–0.85); older age (OR = 0.98, 95% CI 0.97–0.99); and suicidality (OR = 0.76, 95% CI 0.65–0.90).

### Regional variations

Analysis of the details of the three PES regions showed that a history of receiving any psychiatric treatment, whether in-patient, or out-patient, or both was an important factor in the risk of compulsory readmission, but to a slightly lesser extent in the rural area than in urban areas. Whereas ethnicity was relevant specifically in Amsterdam, age was of particular importance in Rotterdam, and level of self-care in Apeldoorn. A subanalysis showed that, independently of patient and process characteristics, PES region was associated with the risk of repeated ECPA. Details are available on request from the authors.

### Factors associated with time to readmission

The Cox regression model showed that lower GAF scores and housing problems were associated with shorter time to compulsory readmission (HR = 1.01, 95% CI 1.00–1.02 and HR = 1.17, 95% CI 1.02–1.34, respectively). It also showed that persistent psychiatric problems were associated with a longer time to compulsory readmission (HR = 0.85, 95% CI 0.73–1.00).

## Discussion

### Main results

In this investigation of the frequency and risk factors for repeated ECPA, we found that 15.6% of patients had two or more ECPAs. Approximately 30% of all ECPAs were repeated ECPAs. The literature reports considerable variations in the frequencies of repeated compulsory psychiatric admission: 5.5% in 7 years (Taiwan);^9^ 36% in 2 years (Switzerland);^10^ and 37% in 5 years (the Netherlands).^8^ Our own finding of 15.6% over 2 years takes account of the different lengths of follow-up, and expresses an average.

We also found that two-thirds of second ECPAs (66.1%) occurred within 6 months of the first. This is similar first to the findings of Lay et al, who reported a peak of compulsory readmissions shortly after discharge;^10^ and also to the findings of van der Post et al, who reported a higher risk of readmission in the first year after discharge than in the subsequent follow-up years.^8^ However, in the first year of follow-up, the study by Lin et al showed relatively low rates of compulsory readmissions.^9^

As well as three static, unmodifiable baseline risk factors for a repeated ECPA – history of receiving any psychiatric treatment whether in-patient, or out-patient, or both, age and ethnicity – we found one relevant factor that could potentially be modified by interventions: level of self-care. With regard to the time to readmission lower GAF scores and housing problems were also potentially modifiable risk factors.

Our findings with regard to history of mental healthcare and age are similar to those in the other studies, in which younger age and an existing treatment history in mental healthcare were consistently identified as important risk factors for repeated ECPAs.^8–10^ Our findings with regard to various factors – level of self-care, housing problems and lower GAF scores – also indicate that patients with severe mental illnesses^14,15^ who show lower level of functioning are particularly susceptible to repeated compulsory psychiatric admissions. This, too, is in accordance with the literature on revolving-door patients.^3–7^

Our finding with regard to ethnicity requires some deliberation. Although one would expect ethnicity other than that of the majority group to be associated with a higher risk of repeated ECPA,^16^ it was clear from the regional differences that ethnicity was important mainly in Amsterdam. This association almost certainly lies in the millions of foreign tourists who visited this city in the period in question. As a result of various psychiatric crises, some of them required the services of the PES and were compulsorily admitted. Once they had returned to their own country, the chance of a repeated ECPA in Amsterdam was close to zero. The effect of these large numbers of short-term foreign visitors probably exceeded any differences in the proportions of Amsterdam's native Dutch residents and its other long-term residents.

### Strengths and limitations

To our knowledge, this study is one of the few to have investigated risk factors for repeated compulsory psychiatric admissions. The database, on which our study was based, had various strengths in its own right. First, it contained a large number of patients and individual PES contacts, and reflected routine daily practice. Second, the EPF was standardised, included a structured assessment scale, and was concise and easy to use. A further strength of the study was the absence of inclusion and exclusion criteria, which provided an advantage with regard to representativeness and generalisability.

There are also some limitations. There were relatively large proportions of missing data on a few database items, such as ethnicity, living situation and substance misuse. A further limitation lay in an effect of our explorative approach, thus, findings do not identify causal factors and relationships but associations – merely suggesting the direction of further research. Also, the clinical data with regard to the compulsory hospital stay and other follow-up treatment after assessment by the PES was not accessible for this study. For example, we were unable to incorporate data on psychotherapeutic and psychopharmacological interventions and determine their relationship with repeated ECPAs. Finally, the odds and hazard ratios were generally relatively small.

### Clinical implications

The importance of preventive measures is demonstrated by the large numbers of patients with an ECPA – and particularly of those with repeated ECPAs – that were found in our study and in earlier studies. On the basis both of earlier findings and of the possible risk factors we identified, interventions to reduce the risk of repeated compulsory psychiatric admissions should focus on improving self-care, general daily functioning and housing problems. To reduce the risk of repeated ECPAs, future studies might then investigate the effects of interventions that specifically target these factors. Examples of these interventions could be found in integrated out-patient treatment programmes, such as flexible assertive community therapy,^17^ which not only focuses on psychopathology but also pays attention to patients’ daily functioning, activities, social network and housing.

Finally, two-thirds of the second ECPAs in our study occurred within 6 months – a very short period. This finding suggests that regardless of their demographic and clinical characteristics, any patient with a recent or very recent ECPA deserves intensive follow-up, and that this should focus on reducing the risk of repeated compulsory psychiatric admission.

## Data Availability

The data that support the findings of this study are available from the corresponding author, M.H.d.J., upon reasonable request.

## Appendix

### Items on the Severity of Psychiatric Illness rating scale

**Table:**

Item	
	*Severity of the problems*
1	Suicide potential
2	Danger to others
3	Severity of psychiatric symptoms
4	Level of self-care
	*Psychiatric complications and comorbidity*
5	Substance abuse
6	Medical complications
7	Family problems
8	Vocational problems
	*Complications in the treatment process*
9	Housing problems
10	Motivation for treatment
11	Medication compliance
12	Illness insight
13	Family involvement
14	Symptom persistence
